# Epidemiology of Human Parechovirus Type 3 Upsurge in 2 Hospitals, Freiburg, Germany, 2018

**DOI:** 10.3201/eid2507.190257

**Published:** 2019-07

**Authors:** Roland Elling, Sindy Böttcher, Florian du Bois, Alexandra Müller, Christiane Prifert, Benedikt Weissbrich, Jörg Hofmann, Klaus Korn, Anna-Maria Eis-Hübinger, Markus Hufnagel, Marcus Panning

**Affiliations:** Medical Center–University of Freiburg Faculty of Medicine, Freiburg, Germany (R. Elling, F. du Bois, A. Müller, M. Hufnagel, M. Panning);; Robert Koch-Institute, Berlin, Germany (S. Böttcher);; University of Würzburg, Würzburg, Germany (C. Prifert, B. Weissbrich);; Charité – Universitätsmedizin Berlin, Berlin (J. Hofmann);; Friedrich Alexander University Erlangen-Nuremberg, Erlangen, Germany (K. Korn);; University of Bonn Medical Centre, Bonn, Germany (A.-M. Eis-Hübinger)

**Keywords:** parechovirus, pediatric, outbreak, viral protein 1 sequence, VP1 sequence, phylogeny, surveillance, viruses, upsurge, Germany

## Abstract

In 2018, a cluster of pediatric human parechovirus (HPeV) infections in 2 neighboring German hospitals was detected. Viral protein 1 sequence analysis demonstrated co-circulation of different HPeV-3 sublineages and of HPeV-1 and -5 strains, thereby excluding a nosocomial outbreak. Our findings underline the need for HPeV diagnostics and sequence analysis for outbreak investigations.

Most human parechovirus (HPeV) infections cause mild upper respiratory tract symptoms or unspecific febrile illnesses. Severe clinical manifestations, such as meningitis/encephalitis, myocarditis, and newborn sepsis are caused by HPeV type 3 (HPeV-3) and have been described in children <3 months of age (*1*). Surveillance data show endemic circulation in several countries, such as the Netherlands (*2*) and the United States (*3*), but studies have discussed the epidemic potential of HPeV-3 in other countries, including Japan (*4*), Australia (*5*), and the United Kingdom (*6*). Nosocomial transmission has been documented (*7*).

However, in most outbreak investigations, determination of HPeV types was performed retrospectively (*7*,*8*). We report on our investigations on a cluster of HPeV infections in 2 neighboring hospitals in Freiburg, Germany. We provide evidence that rapid phylogenetic analysis can assist in outbreak investigations.

## The Study

**Figure 1:**
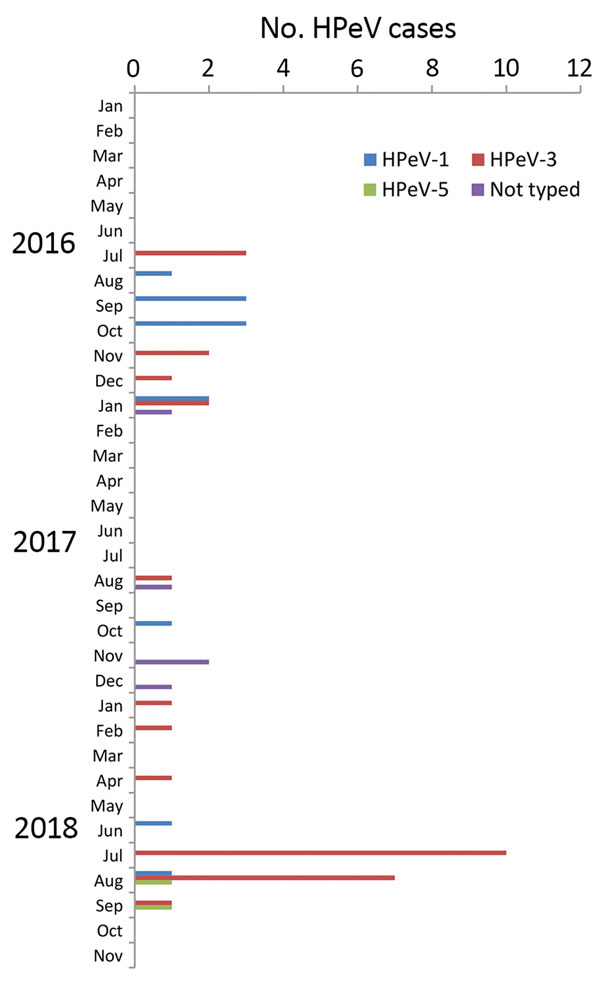
Number of human parechovirus (HPeV) cases in infants and young children by month, Freiburg, Germany, 2016–2018.

During routine diagnostic testing of clinical samples from infants and young children in July 2018, we detected an increase in HPeV cases (Figure 1). We collected >1 clinical specimens from most patients (Table 1). During July 9–August 25, 2018, we documented 19 cases, compared with 4 (2016) and 2 (2017) from this same time span, all using the same assays and diagnostic testing algorithm. In September 2018, only 2 patients tested HPeV-positive; no additional cases were identified during October and November 2018. HPeV diagnostic procedures were performed upon the request of the treating physician. For the detection of HPeV, we used commercial multiplex PCR panels: FTD respiratory pathogens 21 (Fast Track Diagnostics [FTD]; Siemens Healthineers, https://www.siemens-healthineers.com) for respiratory specimens and FTD EPA for cerebrospinal fluid (CSF), plasma, and fecal samples. Patients were hospitalized on 1 ward in hospital A and 4 wards in hospital B (Appendix Figure). We retrieved medical data on HPeV-positive patients from the hospital-based information system. We obtained written informed consent from parents or guardians. 

**Table 1:** Epidemiologic data of human parechovirus cases in Freiburg, Germany, January–September 2018*

Case no.	Patient age, mo/sex	Specimen type				HPeV type
		Cerebrospinal fluid	Upper respiratory tract	Plasma	Feces	
1	2/M	Negative	Positive	NA	Negative	3
2	3/M	Positive	Positive	Positive	Positive	3
3	0/F	Positive	Positive	NA	NA	3
4	19/M	NA	NA	NA	Positive	1
5	1/M	Positive	Positive	Positive	Positive	3
6	1/M	NA	NA	Positive	Positive	3
7	1/F	NA	NA	NA	Positive	3
8	7/M	Negative	NA	NA	Positive	3
9	2/M	Negative	NA	NA	Positive	3
10	2/M	NA	NA	Positive	NA	3
11	1/M	NA	Positive	NA	Positive	3
12	0/M	Positive	Positive	Positive	Positive	3
13	1/M	Positive	Positive	NA	NA	3
14	0/F	Positive	Positive	Positive	Positive	3
15	0/F	NA	NA	Positive	Positive	3
16	2/M	NA	NA	Positive	Positive	3
17	2/M	NA	NA	Positive	Positive	3
18	1/F	NA	NA	Positive	Positive	3
19	17/F	NA	Positive	NA	NA	1
20	1/F	NA	NA	Positive	Positive	3
21	4/F	NA	Positive	NA	Positive	3
22	2/M	Positive	NA	NA	Positive	3
23	0/M	NA	NA	Positive	Positive	5
24	0/M	Positive	NA	NA	Positive	3
25	1/M	NA	NA	Positive	Positive	5

*HPeV, human parechovirus; NA, not applicable (no specimen).

The age of the 2018 HPeV-positive patients ranged from 10 days to 19 months (median 1 month), with 88% of patients being <4 months of age (Table 1). Plasma samples (n = 14) had a diagnostic yield of 100%. The median duration of hospitalization was 4 days (range 3–23 days). The main clinical symptoms of HPeV-3 cases were fever (n = 21; 100%) and poor feeding (n = 16; 76%) (Table 2). None of our patients required admission to a pediatric intensive care unit. All of our patients were discharged from the hospital without complications.

**Table 2:** Clinical symptoms of human parechovirus cases in Freiburg, Germany, January–September 2018*

Clinical signs and symptoms	No. (%) positive patients		
	HPeV-1, n = 2	HPeV-3, n = 21	HPeV-5, n = 2
Fever	1 (50)	21 (100)	2 (100)
Poor feeding	1 (50)	16 (76)	1 (50)
Irritability	0	13 (62)	1 (50)
Rash	1 (50)	6 (29)	1 (50)
Diarrhea	1 (50)	5 (24)	0
Respiratory distress	0	5 (24)	0
Vomiting	1 (50)	0	0

*HPeV, human parechovirus

After we detected the first cases in July 2018, we performed molecular typing of HPeV by amplifying and sequencing the complete viral protein 1 (VP1) genomic region (*9*). Of the 25 HPeV strains detected in Freiburg in 2018, 21 were typed as HPeV-3, 2 were assigned to HPeV-1, and 2 to HPeV-5 (Table 1). This compares with 7 HPeV-1 and 6 HPeV-3 types in Freiburg in 2016, and 3 HPeV-1, 3 HPeV-3, and 5 strains not typed in 2017 (Figure 1).

**Figure 2:**
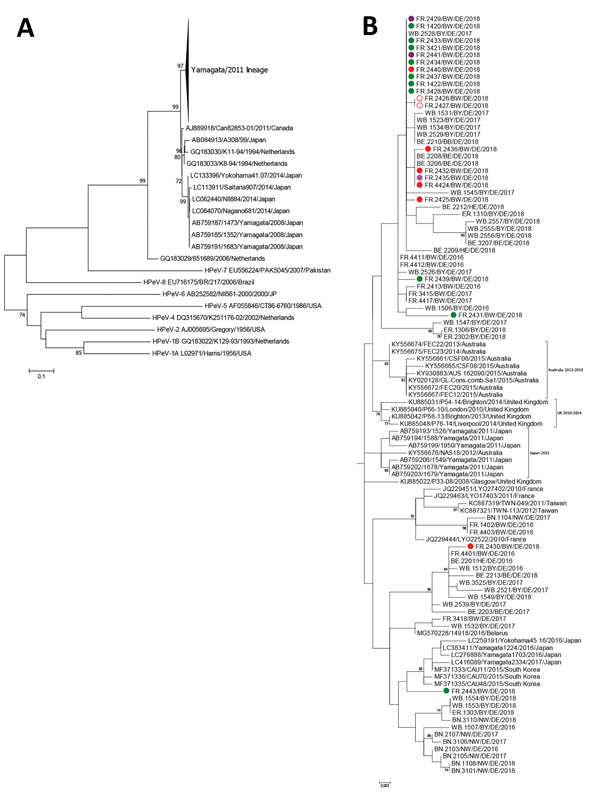
A) Phylogenetic analysis of human parechovirus type 3 strains collected during 2016–2018 from patients hospitalized in Freiburg, Germany, along with strains from 4 geographic regions in Germany based on the viral protein 1 region (678 nt) of the polyprotein gene (n = 74). B) Phylogenetic analysis of Yamagata/2011 parechovirus lineage. Color code depicts wards in the 2 Freiburg hospitals: green, A3; red, B1; pink, B3; purple, B4. Cases in twins are marked with open circles. Scale bars indicate nucleotide substitutions per site.

For phylogenetic analyses, we included HPeV strains detected during January 2016–September 2018 at another 4 university hospitals: Würzburg (n = 56) and Erlangen (n = 10) in southern Germany, Bonn (n = 10) in western Germany, and Charité Berlin (n = 14) in northeastern Germany. We detected 134 HPeV strains in respiratory, fecal, CSF, and serum samples. These were typed based on the VP1 genomic region (*9*). We detected HPeV types 1, 3, 4, 5, and 6. We deposited all sequences in GenBank under accession numbers MK204942–MK204985 and MK291273–MK291362 (Appendix Tables 1,2). For HPeV-3 phylogenetic analysis, we included 74 strains identified during 2016–2018 and compared them with representative reference strains available from GenBank (Figure 2). Because of high nucleotide variability in the 3′ end of the VP1 coding region, we included only complete VP1 sequences.

As recently described, 2 HPeV-3 lineages have been identified (*10*). Widespread clustering proved co-circulation of the 2016–2018 HPeV strains from Germany (Figure 2). One cluster comprising German strains was most closely related to HPeV-3 identified in Japan (98.82% nt identity), Australia (98.82%), and the United Kingdom (99.12%).

Among the 21 Freiburg 2018 HPeV-3 strains, 3 groups of completely identical VP1 sequences (10, 3, and 2 sequences) were observed. However, no separate clustering could be detected among these strains because HPeV sequences from other regions in Germany also were assigned to these groups. A direct epidemiologic link could be drawn between 2 cases (cases 12 and 14, with completely identical VP1 sequences, were in twins; Figure 2). Another 2 cases (cases 15 and 18) shared time on the same ward and also displayed 100% identical sequences. However, no hospital ward–specific clustering was observed, suggesting community-acquired transmission.

## Conclusions

Routine diagnostics showed an unexpectedly high number of HPeV cases during a 6-week period in 2 neighboring hospitals in Freiburg, Germany. This raised concern about the possibility of a nosocomial outbreak. Recently, healthcare-associated transmission of HPeV-3 has been described. This makes timely identification of outbreaks essential from a hospital hygiene, as well as a public health, perspective (*7*).

Several patients showed signs of sepsis-like illness, including the clinical triad of fever, poor feeding, and irritability. This is similar to a UK case series reporting a cluster of HPeV infections among infants in 2016 (*6*). In our study, HPeV-3 was detected exclusively in CSF samples, indicating a more severe clinical phenotype compared with HPeV-1 and -5 infections, supporting previous data (*11*). Studies have shown that rapid detection of HPeV reduced length of hospital stay and antimicrobial drug use. This emphasizes the usefulness of HPeV diagnostics (*1*). We showed that HPeV diagnostics, including molecular typing, helped to exclude a nosocomial outbreak. Diagnostically, plasma, respiratory swab, and fecal samples all showed high detection rates, and most patients were positive in >1 area. Testing of blood samples for enterovirus detection was recently proposed for infants and should be considered for HPeV accordingly (*12*).

We demonstrated different HPeV types and sublineages, including 2 rare HPeV-5 infections. By conducting phylogenetic analysis in combination with reviewing epidemiologic data, we could exclude a nosocomial outbreak. However, based on this information, transmissions could not be ruled out in 2 independent events with 2 cases each. Although a cluster of HPeV-3 infections has been described (*6*), retrospective sequence analysis showed different clustering of the identified strains (*13*). Because of low nucleotide variability, sequence-based differentiation between HPeV-3 strains remains ambiguous, a circumstance that impedes molecular outbreak investigation (*14*).

Our study has limitations. There is a lack of available sequence data from pediatric patients in Germany. In contrast to reports from the Netherlands and the UK, a biannual cycle of HPeV infections has not been demonstrated in Germany; however, our data suggest a biannual cycle. From a public health perspective, a central repository for HPeV sequences, together with key anonymized clinical data from human cases, would improve our understanding of HPeV epidemiology and virus evolution. Institutionalized surveillance similar to the enterovirus surveillance and typing systems already in place across Europe could serve as a blueprint (*8*,*15*).

Our report underscores the usefulness of HPeV diagnostics in infants. It illustrates the power of VP1 sequence–guided phylogenetic HPeV analysis, which helped, in combination with epidemiologic data, to rapidly investigate an HPeV outbreak.

Appendix Additional information on human parechovirus cases in Germany, 2018.
