# Supplementary material for: Epidemiology of Human Parechovirus Type 3 Upsurge in 2 Hospitals, Freiburg, Germany, 2018
Source: Emerg Infect Dis. 2019 Jul;25(7):1384–8. doi: 10.3201/eid2507.190257 (PMC6590756; doi:10.3201/eid2507.190257)
Supplement: Appendix — Additional information on human parechovirus cases in Germany, 2018. [file 19-0257-Techapp-s1.pdf]

Article DOI: https://doi.org/10.3201/eid2507.190257

Molecular Epidemiology of Human Parechovirus Type 3 Upsurge in 2 Hospitals, Freiburg, Germany, 2018

Appendix

Appendix Table 1. Sample type, GenBank accession number, and strain ID of human parechovirus cases, Freiburg, Germany, January–September 2018*

Case no.	Sample type	Accession no.	Strain ID
1	Upper respiratory tract	MK204975	FR.1420/BW/DE/2018
2	CSF	MK204964	FR.3421/BW/DE/2018
3	Upper respiratory tract	MK204969	FR.1422/BW/DE/2018
4	Fecal	MK204944	FR.2423/BW/DE/2018
5	Fecal	MK204971	FR.2429/BW/DE/2018
6	Fecal	MK204980	FR.2430/BW/DE/2018
7	Fecal	MK204976	FR.2431/BW/DE/2018
8	Fecal	MK204970	FR.2432/BW/DE/2018
9	Fecal	MK204956	FR.2433/BW/DE/2018
10	Plasma	MK204954	FR.4424/BW/DE/2018
11	Fecal	MK204958	FR.2425/BW/DE/2018
12	Fecal	MK204962	FR.2427/BW/DE/2018
13	CSF	MK204963	FR.3428/BW/DE/2018
14	Fecal	MK204957	FR.2426/BW/DE/2018
15	Fecal	MK204966	FR.2434/BW/DE/2018
16	Fecal	MK204955	FR.2435/BW/DE/2018
17	Fecal	MK204972	FR.2436/BW/DE/2018
18	Fecal	MK204968	FR.2437/BW/DE/2018
19	Upper respiratory tract	MK204951	FR.1438/BW/DE/2018
20	Fecal	MK204967	FR.2440/BW/DE/2018
21	Fecal	MK204977	FR.2439/BW/DE/2018
22	Fecal	MK204965	FR.2441/BW/DE/2018
23	Fecal	MK204943	FR.2442/BW/DE/2018
24	Fecal	MK204979	FR.2443/BW/DE/2018
25	Plasma	MK204942	FR.4444/BW/DE/2018

*CSF, cerebrospinal fluid; ID, identification.

Appendix Table 2. GenBank accession number, strain ID, specimen type, year of sampling and human parechovirus (HPeV) type of cases in Bonn (BN), Charité Berlin (BE), Erlangen (ER), Freiburg (FR; 2016–2017 only), and Würzburg (WB), Germany, January 2016–September 2018

Accession no.	Strain ID	Specimen type	Year	HPeV type
MK291287	BN.3101/NW/DE/2018	Cerebrospinal fluid	2018	3
MK291288	BN.1102/NW/DE/2016	Respiratory	2016	1
MK291291	BN.2103/NW/DE/2016	Fecal	2016	3
MK291289	BN.1104/NW/DE/2017	Respiratory	2017	3
MK291292	BN.2105/NW/DE/2017	Fecal	2017	3
MK291295	BN.3106/NW/DE/2017	Cerebrospinal fluid	2017	3
MK291293	BN.2107/NW/DE/2017	Fecal	2017	3
MK291290	BN.1108/NW/DE/2018	Respiratory	2018	3
MK291294	BN.2109/NW/DE/2018	Fecal	2018	1
MK291296	BN.3110/NW/DE/2018	Cerebrospinal fluid	2018	3
MK291275	BE.2201/HE/DE/2016	Fecal	2016	3
MK291273	BE.1202/BE/DE/2016	Respiratory	2016	1
MK291276	BE.2203/BE/DE/2017	Fecal	2017	3
MK291286	BE.4204/BE/DE/2018	Serum	2018	6
MK291274	BE.1205/BE/DE/2018	Respiratory	2018	4
MK291284	BE.3206/BE/DE/2018	Cerebrospinal fluid	2018	3
MK291285	BE.3207/BE/DE/2018	Cerebrospinal fluid	2018	3
MK291277	BE.2208/BE/DE/2018	Fecal	2018	3
MK291278	BE.2209/HE/DE/2018	Fecal	2018	3
MK291279	BE.2210/BB/DE/2018	Fecal	2018	3
MK291280	BE.2211/HE/DE/2018	Fecal	2018	5
MK291281	BE.2212/HE/DE/2018	Fecal	2018	3
MK291282	BE.2213/BE/DE/2018	Fecal	2018	3
MK291283	BE.2214/HE/DE/2018	Fecal	2018	4
MK291297	ER.1301/BY/DE/2018	Respiratory	2018	5
MK291303	ER.2302/BY/DE/2018	Fecal	2018	3
MK291298	ER.1303/BY/DE/2018	Respiratory	2018	3
MK291304	ER.2304/BY/DE/2018	Fecal	2018	3
MK291305	ER.2305/BY/DE/2018	Fecal	2018	5
MK291299	ER.1306/BY/DE/2018	Respiratory	2018	3
MK291300	ER.1307/BY/DE/2018	Respiratory	2018	1
MK291301	ER.1308/BY/DE/2018	Respiratory	2018	1
MK291306	ER.2309/BY/DE/2018	Fecal	2018	5
MK291302	ER.1310/BY/DE/2018	Respiratory	2018	3
MK204981	FR.4401/BW/DE/2016	Plasma	2016	3
MK204985	FR.1402/BW/DE/2016	Respiratory	2016	3
MK204983	FR.4403/BW/DE/2016	Plasma	2016	3
MK204984	FR.2404/BW/DE/2016	Fecal	2016	1
MK204950	FR.1405/BW/DE/2016	Respiratory	2016	1
MK204982	FR.1406/BW/DE/2016	Respiratory	2016	1
MK204949	FR.1407/BW/DE/2016	Respiratory sample	2016	1
MK204948	FR.2408/BW/DE/2016	Fecal	2016	1
MK204953	FR.1409/BW/DE/2016	Respiratory	2016	1
MK204947	FR.2410/BW/DE/2016	Fecal	2016	1
MK204960	FR.4411/BW/DE/2016	Plasma	2016	3
MK204959	FR.4412/BW/DE/2016	Plasma	2016	3
MK204961	FR.2413/BW/DE/2016	Fecal	2016	3
MK204946	FR.1414/BW/DE/2017	Respiratory	2017	1
MK204973	FR.3415/BW/DE/2017	Cerebrospinal fluid	2017	3
MK204952	FR.1416/BW/DE/2017	Respiratory	2017	1
MK204974	FR.4417/BW/DE/2017	Plasma	2017	3
MK204978	FR.3418/BW/DE/2017	Cerebrospinal fluid	2017	3
MK204945	FR.1419/BW/DE/2017	Respiratory	2017	1
MK291307	WB.1501.BY/2016/DEU	Respiratory	2016	1
MK291308	WB.1502.BY/2016/DEU	Respiratory	2016	1
MK291309	WB.1503.BY/2016/DEU	Respiratory	2016	1
MK291310	WB.1504.BY/2016/DEU	Respiratory	2016	1
MK291311	WB.1505.BY/2016/DEU	Respiratory	2016	1
MK291312	WB.1506.BY/2016/DEU	Respiratory	2016	3

Accession no.	Strain ID	Specimen type	Year	HPeV type
MK291313	WB.1507.BY/2016/DEU	Respiratory	2016	3
MK291314	WB.1508.BY/2016/DEU	Respiratory	2016	1
MK291315	WB.1509.BY/2016/DEU	Respiratory	2016	4
MK291316	WB.1510.BY/2016/DEU	Respiratory	2016	1
MK291317	WB.1511.BY/2016/DEU	Respiratory	2016	1
MK291318	WB.1512.BY/2016/DEU	Respiratory	2016	3
MK291319	WB.1513.BY/2016/DEU	Respiratory	2016	1
MK291320	WB.1514.BY/2016/DEU	Respiratory	2016	1
MK291321	WB.1515.BY/2016/DEU	Respiratory	2016	1
MK291322	WB.1516.BY/2017/DEU	Respiratory	2017	1
MK291323	WB.1517.BY/2017/DEU	Respiratory	2017	4
MK291324	WB.1518.BY/2017/DEU	Respiratory	2017	3
MK291325	WB.1519.BY/2017/DEU	Respiratory	2017	1
MK291326	WB.1520.BY/2017/DEU	Respiratory	2017	1
MK291354	WB.2521.BY/2017/DEU	Fecal	2017	3
MK291327	WB.1522.BY/2017/DEU	Respiratory	2017	1
MK291328	WB.1523.BY/2017/DEU	Respiratory	2017	3
MK291329	WB.1524.BY/2017/DEU	Respiratory	2017	1
MK291362	WB.3525.BY/2017/DEU	Cerebrospinal fluid	2017	3
MK291355	WB.2526.BY/2017/DEU	Fecal	2017	3
MK291330	WB.1527.BY/2017/DEU	Respiratory	2017	1
MK291356	WB.2528.BY/2017/DEU	Fecal	2017	3
MK291357	WB.2529.BY/2017/DEU	Fecal	2017	3
MK291331	WB.1530.BY/2017/DEU	Respiratory	2017	1
MK291332	WB.1531.BY/2017/DEU	Respiratory	2017	3
MK291333	WB.1532.BY/2017/DEU	Respiratory	2017	3
MK291334	WB.1533.BY/2017/DEU	Respiratory	2017	1
MK291335	WB.1534.BY/2017/DEU	Respiratory	2017	3
MK291336	WB.1535.BY/2017/DEU	Respiratory	2017	1
MK291337	WB.1536.BY/2017/DEU	Respiratory	2017	6
MK291338	WB.1537.BY/2017/DEU	Respiratory	2017	1
MK291339	WB.1538.BY/2017/DEU	Respiratory	2017	6
MK291358	WB.2539.BY/2017/DEU	Fecal	2017	3
MK291340	WB.1540.BY/2017/DEU	Respiratory	2017	1
MK291341	WB.1541.BY/2017/DEU	Respiratory	2017	6
MK291342	WB.1542.BY/2017/DEU	Respiratory	2017	4
MK291343	WB.1543.BY/2017/DEU	Respiratory	2017	6
MK291344	WB.1544.BY/2017/DEU	Respiratory	2017	6
MK291345	WB.1545.BY/2017/DEU	Respiratory	2017	3
MK291346	WB.1547.BY/2017/DEU	Respiratory	2017	3
MK291347	WB.1548.BY/2018/DEU	Respiratory	2018	1
MK291348	WB.1549.BY/2018/DEU	Respiratory	2018	3
MK291349	WB.1550.BY/2018/DEU	Respiratory	2018	6
MK291350	WB.1551.BY/2018/DEU	Respiratory	2018	6
MK291351	WB.1552.BY/2018/DEU	Respiratory	2018	1
MK291352	WB.1553.BY/2018/DEU	Respiratory	2018	3
MK291353	WB.1554.BY/2018/DEU	Respiratory	2018	3
MK291359	WB.2555.BY/2018/DEU	Fecal	2018	3
MK291360	WB.2556.BY/2018/DEU	Fecal	2018	3
MK291361	WB.2557.BY/2018/DEU	Fecal	2018	3

*ID, identification.

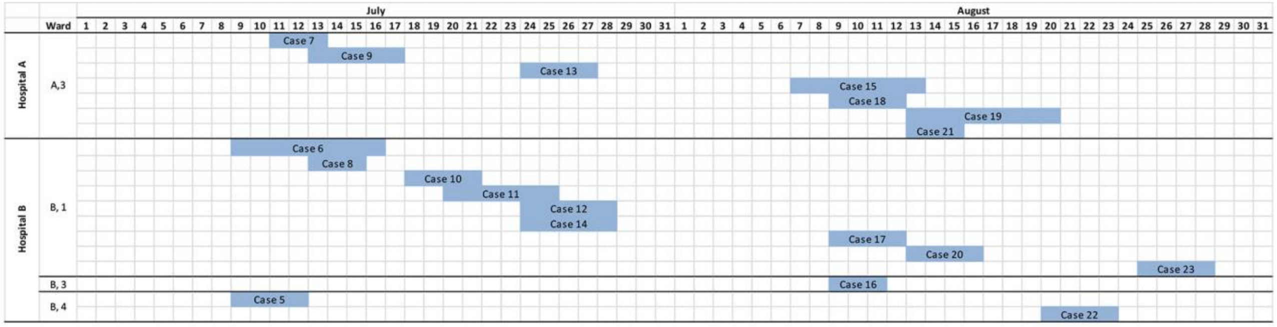

Appendix Figure. Length of stay on assigned wards of 19 patients with human parechovirus, Freiburg, Germany, July 9–August 25, 2018.
